# Disability, violence, and mental health among Somali refugee women in a humanitarian setting

**DOI:** 10.1017/gmh.2020.23

**Published:** 2020-10-29

**Authors:** Mazeda Hossain, Rachel Pearson, Alys McAlpine, Loraine Bacchus, Sheru W. Muuo, Stella K Muthuri, Jo Spangaro, Hannah Kuper, Giorgia Franchi, Ricardo Pla Cordero, Sarah Cornish-Spencer, Tim Hess, Martin Bangha, Chimaraoke Izugbara

**Affiliations:** 1 Department of Global Health and Development, London School of Hygiene and Tropical Medicine, UK; 2 Centre for Women, Peace & Security, London School of Economics and Political Science, UK; 3 Population, Policy and Practice Research and Teaching Department, UCL Great Ormond Street Institute of Child Health, UK; 4 African Population and Health Research Center, Kenya; 5 School of Health and Society, University of Wollongong, Australia; 6 International Rescue Committee, UK

**Keywords:** Disability, humanitarian crisis, mental health, refugees, violence

## Abstract

**Background:**

There is limited evidence on the relationship between disability, experiences of gender-based violence (GBV), and mental health among refugee women in humanitarian contexts.

**Methods:**

A cross-sectional analysis was conducted of baseline data (*n* = 209) collected from women enrolled in a cohort study of refugee women accessing GBV response services in the Dadaab refugee camps in Kenya. Women were surveyed about GBV experiences (past 12 months, before the last 12 months, before arriving in the refugee camps), functional disability status, and mental health (anxiety, depression, post-traumatic stress), and we explored the inter-relationship of these factors.

**Results:**

Among women accessing GBV response services, 44% reported a disability. A higher proportion of women with a disability (69%) reported a past-year experience of physical intimate partner violence and/or physical or sexual non-partner violence, compared to women without a disability (54%). A higher proportion of women with a disability (32%) experienced non-partner physical or sexual violence before arriving in the camp compared to women without a disability (16%). Disability was associated with higher scores for depression (1.93, 95% confidence interval (CI) 0.54–3.33), PTSD (2.26, 95% CI 0.03–4.49), and anxiety (1.54, 95% CI 0.13–2.95) after adjusting for age, length of encampment, partner status, number of children, and GBV indicators.

**Conclusions:**

A large proportion of refugee women seeking GBV response services have disabilities, and refugee women with a disability are at high risk of poor mental health. This research highlights the need for mental health and disability screening within GBV response programming.

## Introduction

It is estimated that one billion people – or 15% of the global population – live with a disability (World Health Organization, 2002; Rohwerder, 2015) and that this prevalence is even higher in humanitarian settings (International Centre for Evidence in Disability (ICED), 2019). There is growing evidence that women and girls with disabilities are at an increased risk of experiencing violence throughout their life cycle (Devries *et al*., 2018). Within humanitarian settings, violence against women is common and has been shown to increase during and after periods of conflict (Hossain *et al*., 2014b; Murphy *et al*. 2019). However, there is limited evidence on the prevalence or correlates of gender-based violence (GBV) among refugees with disabilities (Marshall and Barrett, 2018).

The humanitarian community has defined GBV as an umbrella term for any harmful act that is perpetrated against a person's will and is based on socially ascribed (i.e. gender) differences between females and males (UNFPA, 2015). GBV includes acts of physical, sexual, or mental harm or suffering, threats, coercion, and other deprivations of liberty and may be perpetrated by an intimate partner or non-partner. The health consequences of violence are well-established; GBV can lead to physical impairment or injury and has both short- and long-term effects on an individual's psychological well-being (Dillon *et al*., 2013; Satyanarayana *et al*., 2015). Among conflict-affected populations, these physical and psychological health consequences linger well beyond the emergency period and often impact on an individual's ability to function in the post-conflict stage of a crisis (Usta *et al*., 2008; Hustache *et al*., 2009; Betancourt *et al*., 2010; Roberts and Browne, 2011; Dossa *et al*., 2014; Hossain *et al*., 2014b). Furthermore, exposure to torture, including violence and other war-related traumatic events, is associated with higher rates of post-traumatic stress disorder (PTSD), depression, anxiety, and suicidal thoughts or attempts (Campbell and Lewandowski, 1997; Fazel *et al*., 2005; Hunt and Gakenyi, 2005; Pico-Alfonso *et al*., 2006; Johnson and Thompson, 2008; Steel *et al*., 2009; Tol *et al*., 2010; Beydoun *et al*., 2012; McLaughlin *et al*., 2012; Kalt *et al*., 2013; Kouyoumdjian *et al*., 2013; Ba and Bhopal, 2017).

Certain groups may be particularly vulnerable to GBV, and among these are people with disabilities, who make up 15% of the global population (World Health Organization, 2002; Kostanjsek, 2011; World Health Organization (WHO) and The World Bank, 2011). Disability, as defined by The International Classification of Functioning, Disability and Health (ICF), is complex and is experienced at the level of impairment, activity limitations, and participation restriction (World Health Organization, 2002). The ICF model recognises the interaction of an individual's functional status with personal factors and physical, cultural, and policy environmental factors in creating disability (Kostanjsek, 2011).

There is growing evidence that people with disabilities are more vulnerable to violence (Puri *et al*., 2015; Gupta *et al*., 2018), with recent research suggesting that women with disabilities within low- and middle-income countries are two to four times more likely to experience intimate partner violence (IPV) compared to women without disabilities (Hughes *et al*., 2012; Dunkle *et al*., 2018; Stern *et al*., 2020). Other research has found significant correlations between disability and poor mental health outcomes including anxiety and depression (Kinne *et al*., 2004; Dembo *et al*., 2018). However, there is little evidence from humanitarian and conflict settings and limited trial data (Hughes *et al*., 2012; Jones *et al*., 2012; Mikton *et al*., 2014; Sipsma *et al*., 2015; Devries *et al*., 2018; Scolese *et al*., 2020*a*, *b*). Emerging research from low- and middle-income countries is starting to explore the links between disability, gender, and violence (Dunkle *et al*., 2018). A recent meta-analysis of research conducted across six countries (Afghanistan, Bangladesh, Ghana, Nepal, South Africa, and Tajikistan) using data from 4500 women found disability may increase a woman's risk of experiencing non-partner sexual violence; and women with severe disabilities are at greater risk of experiencing both IPV and non-partner sexual violence. In addition, increased stigma and discrimination experienced by women with disabilities may further reduce their ability to access help (Dunkle *et al*., 2018). People with disabilities may be more vulnerable to violence because of their marginalised position in society which can include the need for regular assistance, discrimination, and physical and communication barriers. This may, in turn, impact their ability to disclose abuse and access support (Nosek *et al*., 2001; Scolese *et al*., 2020a). This may also make them less resilient to cope with the impact of GBV, and therefore potentially more likely to suffer adverse mental health consequences, although data are lacking (Brütt *et al*. 2013; Linden, 2017).

The humanitarian sector has developed protection and response programmes to address the physical and psychological health needs of violence survivors and often separately, programmes to address the needs of people with disabilities. However, these two sectors are still in the nascent stage and the programming and evidence base rarely overlap, leaving large gaps in understanding how these services can become more inclusive and best meet the specialised needs of survivors with disabilities (Mirza, 2015; Shaw and Funk, 2019; Stern *et al*., 2020). This gap in the evidence base is noteworthy and understanding this intersection – disability, gender, violence, and mental health – is urgently required to address the needs of violence survivors with disabilities so that they may have equal opportunity to access appropriate services that meet their needs.

This analysis sought to understand the relationship between disability, experiences of GBV, and mental health among refugee women and adolescent girls in a humanitarian context, using baseline data collected from a cohort study of refugee women accessing GBV services in the Dadaab refugee camps in Kenya. Understanding this intersection can inform recommendations for GBV response strategies and programming that meet the needs of refugee women with disabilities. At the time of data collection (2016), the Dadaab refugee complex was the largest and one of the oldest refugee camps in the world (UNHCR, 2017).

## Methods

### Study design, target population, and eligibility criteria

This paper uses baseline data from a prospective cohort study that aimed to explore the feasibility and acceptability of GBV response services using case management and task sharing in the Dadaab refugee camps. Data were collected between February and November 2016.

The study was jointly led by the London School of Hygiene & Tropical Medicine and the African Population Health Research Center.

Women and adolescent girls over the age of 15 years old were recruited from the GBV response centres in two camps within Dadaab (Dagahaley and Hagadera) run by two humanitarian NGOs (International Rescue Committee and Care Kenya). Adolescent girls between the ages of 15–17 years old were eligible to participate if they were the sole head of their household. Eligibility to participate in the study was assessed by each centre's staff during the normal centre intake process. A total of 209 women (132 from Hagadera, 77 from Dagahaley) were enrolled in the cohort and subsequently completed the baseline questionnaire conducted by a trained member of the research field team with interviews taking approximately 1 hour. No women under the age of 18 accessed GBV services during the study period therefore the analysis is limited women who were at least 18 years old at intake. Further study details can be found in the main study report (Hossain *et al*., 2018).

### Context

In 2017, the Dadaab refugee complex hosted 246 551 refugees. It was initially created in 1991 to host Somalis fleeing the Somali Civil War, who at the time of data collection formed the majority of refugees (UNHCR, 2017). Within the camps, GBV response services are delivered through support centres utilising a case management service model with task-sharing components. Services were available to anyone within private spaces run, separate from the community at large, but still located within broader service centres so that GBV services could be accessed without stigma. In addition to response services, the GBV support centres also provided outreach and camp-based violence prevention activities. Additional details on the GBV services are available elsewhere (Hossain *et al*., 2018; Izugbara *et al*., 2018; McAlpine *et al*., 2020; Muuo *et al*., 2020).

### Ethics

The Ethics Committee at the London School of Hygiene & Tropical Medicine (LSHTM Ethics Ref: 8909) and the Scientific Review Committee of the African Medical and Research Foundation (Protocol Reference Number P173-2015) approved the study in 2015. The study was also reviewed and approved by the UN High Commission on Refugees in Kenya. Ethical research procedures were established to ensure that participation in the research did not further traumatise or burden the research participants, the GBV centre staff, or field researchers (World Health Organization, 2016; Hossain and McAlpine, 2017). All participants provided informed consent, which included an acknowledgement that their participation was voluntary and would not influence their access to any services within the camp. No monetary compensation was provided.

This research was developed without the research participants' direct involvement due to the sensitivity of the research and to avoid further traumatisation. Instead, we engaged with GBV response centre case workers, refugee community workers (who were also survivors of violence similar to the research participants), and other field and technical staff who work on direct service provision, to ensure that the development of the research questions and outcome measures were informed by the GBV survivors' priorities, experience, and preferences. This collaboration included contributions throughout all stages of the research from designing the survey tool, determining selection criteria, recruitment , supporting data interpretation and dissemination. Dissemination of the research findings within the Dadaab refugee camps occurred at two stages – after the preliminary data analysis was completed and again after the study was completed. Additional details on the collaborative design process are available (Hossain et al., 2018, McAlpine et al., 2020).

### Survey tool development

The baseline questionnaire was developed using an iterative approach that aimed to limit the time burden on participants. A questionnaire developed for a survey conducted with refugee community workers was modified and refined for the survivor cohort study. An earlier phase of the research with refugee community workers tested sensitive questions – on violence, migration history, and potentially difficult to translate questions such as mental health scales items – in a cross-sectional survey with refugee community workers in the same camps (Hossain *et al*., 2018). The surveys were developed in English, then translated to Somali, and finally back-translated to English. This allowed interviews to be conducted in either language without the use of interpreters, with translation carried out by a group of field researchers fluent in Somali and English.

## Measures

### Gender-based violence

Experiences of non-partner violence (NPV) and IPV were captured in the survey. Seventeen items adapted from the World Health Organization's multi-country study on women's health and domestic violence against women (García-Moreno *et al*., 2005) were used to record reports of emotional, physical and sexual IPV and physical and sexual NPV within several time periods of interest including the past year and prior to camp arrival. IPV was identified among ever-partnered participants who were asked whether their current or most recent partner had perpetrated specific acts of emotional, physical, and sexual violence against them within the time period of interest. Emotional IPV included any instance of a woman's partner: (a) became angry when she spoke to other men; (b) insisted on knowing where she was at all times; (c) forbade her from seeing friends; (d) acted in a frightening or intimidating way; or (e) threatened the use of violence. Physical IPV was recorded where individuals experienced two or more acts of physical violence (slapped, having something thrown at you, pushed or hit with a hand or other object) or any act of severe physical violence (kicked, dragged, beaten, choked, burned intentionally, threatened or assaulted with a gun/knife/other weapons) perpetrated by their partner, as is consistent with other investigations using these items (Hossain *et al*., 2014a). Sexual IPV was defined as any experience of forced sex; this includes forced sex via threats and intimidation. Physical NPV was recorded where individuals reported being: (1) beaten with a fist, kicked, or hurt with an object; and (2) assaulted with a gun, knife, or other weapons by a non-partner. Sexual NPV was again defined as any act of forced sex. In addition to measuring the time period when the violence occurred (past year, before arriving in Dadaab), women were also asked about perpetrator types for all NPV experiences (i.e. combatant, neighbour, family member). Time period and perpetrator type were used to examine conflict- or war-related violence.

### Disability

The Washington Group Short Set (WG-SS) of disability questions was used to identify women with a disability at their baseline interview, as measured by self-reported difficulty functioning (Madans *et al*., 2004; Washington Group on Disability Statistics, 2019). Six core functional domains are addressed: walking, seeing, hearing, cognition, self-care, and communication. Women were asked to report if they have difficulties in each of these six domains (reported as ‘none’, ‘some’, ‘a lot of difficulty’ or ‘cannot do at all’), and thereby these questions have been designed to identify the majority of people who are at risk of participation restrictions (Madans *et al*., 2004). In our analysis, women answering ‘a lot of difficulty’ or ‘cannot do at all’ to at least one question were considered to have a disability.

### Mental health outcomes

Three symptomatic scales were used to assess anxiety, depression, and PTSD in the two weeks prior to the interview among the cohort. Each scale was scored on a 4-point Likert scale, with higher scores indicating greater severity.

Anxiety was measured using the generalised anxiety disorder assessment (GAD-7), a 7-item anxiety scale used to screen for generalised anxiety disorder (Spitzer *et al*., 2006). Cut-off scores of 5, 10, and 15 indicate mild, moderate, and severe anxiety, respectively.

Depression was measured using the patient health questionnaire (PHQ-9), a 9-item scale based upon the depression criteria from the diagnostic and statistical manual for mental disorders (DSM- IV) (Kroenke *et al*., 2001). Cut-off scores of 5, 10, 15, and 20 indicate mild, moderate, moderately severe, and severe depression, respectively. A previous study that translated the PHQ-9 to Somali found that it had good internal validity among Somali immigrants living in the US (Nallusamy *et al*., 2016).

PTSD was measured using the post-traumatic symptom subscale of the Harvard Trauma Questionnaire (HTQ-PTSD). The HTQ-PTSD is a 16-item sub-scale derived from the DSM-IV PTSD criteria (Mollica *et al*., 1992). This screening tool for probable PTSD, developed for adult refugees, has been validated within several refugee populations (Mollica *et al*., 1992; Silove *et al*., 2014). The PTSD scale has previously been used among Somali refugees in Melkadid camp, Ethiopia, demonstrating good internal validity (Feyera *et al*., 2015). A mean score cut-off of 2 (from a theoretical range of 1–4) has been established to identify probable PTSD.

### Socio-demographic characteristics

The socio-demographic characteristics assessed included continuous measures: age at interview (years), average monthly income (KES), length of encampment (years), and number of children; and categorical measures: literacy status (can read and write *v.* cannot), nationality (Somali *v.* all others), religion (Muslim *v.* Christian), partner status (partner present *v.* partner absent/no current partner), substance abuse by a partner, and belonging to a majority Somali clan (Darood, Dir, Hawiye or Isaaq clans *v.* all others).

### Statistical analysis

Descriptive statistics of socio-demographic characteristics, violence exposures, disability domains, and mental health symptomatic scores for anxiety, depression, and PTSD were produced for all women in the cohort (*n* = 209). Due to the small sample size, violence exposures were aggregated into perpetrator groups: IPV which included physical, sexual, and emotional violence, and NPV which included physical and sexual violence. Frequencies of characteristics among the cohort are presented stratified by disability status. Due to the small sample size, we are unable to make inferences about the population the cohort is sampled from therefore descriptive statistics without *p* values are presented.

The association between having a disability and experiencing different forms of GBV was modelled using fixed-intercept logistic regression, allowing the intercept to vary by the camp to account for camp-level clustering. The association between having a disability and mental health conditions – anxiety, depression, and PTSD – was modelled via fixed-intercept linear regression models. Robust standard errors for model coefficients were estimated using the sandwich estimator. Age, length of encampment, partner status, number of children, and exposure to violence were selected *a priori* for inclusion in the adjusted model, as all are important predictors of both mental health and disability, and may confound the relationship between disability and mental health. We also explored experiences of different forms of GBV as mediators (and likely confounders) in the relationship between disability and mental health.

Models excluded five participants who were missing data on the length of their encampment at baseline (*n* = 204). Model assumptions were checked using residual plots. Due to the small sample size, no interaction terms were considered (Leon and Heo, 2009). The magnitude and direction of model coefficients and their accompanying 95% confidence interval (CI) were considered, and analysis was conducted using Stata v15.

## Results

### Socio-demographic characteristics and prevalence of disability

Women in the sample were aged between 18 and 69 years old. The majority of women identified as Somali (94%) and Muslim (99%) and the median age they first lived with a male partner was 16 years old. The median length of the encampment was 9 years, and 18 women (9%) were born in Dadaab. Most of the women were born in South Central Somalia (86%). There was little observed difference in the county of birth, nationality, religion, years in the camp, and age at the first partnership between women with and without a disability (Table 1).

**Table 1:** Demographics and prevalence of violence by disability status

Baseline characteristics		No disability (*N* = 118)	Disability (*N* = 91)	Total (*N* = 209)
Age [median (IQR)]		25.00 [21.00–30.75]	30.00 [24.00–37.50]	26.00 [22.00–34.00]
Length of encampment (%)	<10 years	63 (53.4)	45 (49.5)	108 (51.7)
	10+ years	42 (35.6)	36 (39.6)	78 (37.3)
	Born in Dadaab	8 (6.8)	10 (11.0)	18 (8.6)
	Missing	5 (4.2)	0 (0.0)	5 (2.4)
Length of encampment [median (IQR)]		8.00 [6.00–17.00]	10.00 [7.00–20.00]	9.00 [6.00–18.00]
Nationality (%)	Other	9 (7.6)	3 (3.3)	12 (5.7)
	Somalian	109 (92.4)	88 (96.7)	197 (94.3)
Birthplace (%)	Dadaab	8 (6.8)	10 (11.0)	18 (8.6)
	Others	8 (6.8)	2 (2.2)	10 (4.8)
	Somalia	102 (86.4)	79 (86.8)	181 (86.6)
Religion (%)	Christian	3 (2.5)	0 (0.0)	3 (1.4)
	Muslim	115 (97.5)	91 (100.0)	206 (98.6)
Partner status (%)	No current partner	60 (50.8)	55 (60.4)	115 (55.0)
	Partner, living together	37 (31.4)	23 (25.3)	60 (28.7)
	Partner, not living together	21 (17.8)	13 (14.3)	34 (16.3)
Children (%)	None	26 (22.0)	16 (17.6)	42 (20.1)
	1–3	48 (40.7)	34 (37.4)	82 (39.2)
	4+	44 (37.3)	41 (45.1)	85 (40.7)
Pregnant over follow-up (%)		37 (31.4)	12 (13.2)	49 (23.4)
Can read and write (%)		53 (44.9)	27 (29.7)	80 (38.3)
Income (KES) (%)	0–4499	83 (70.3)	44 (48.4)	127 (60.8)
	4500–9999	19 (16.1)	32 (35.2)	51 (24.4)
	10 000+	16 (13.6)	15 (16.5)	31 (14.8)
Income (KES) (median (IQR))		3000.00 [0.00–6000.00]	5000.00 [1500.00–6000.00]	3000.00 [80.00–6000.00]
Employment in camp (%)		43 (36.4)	41 (45.1)	84 (40.2)
Age first lived with partner [median (IQR)]		16.00 [14.00–18.00]	16.00 [14.75–18.00]	16.00 [14.00–18.00]
Substance abuse by partner (%)^a^		32 (55.2)	25 (69.4)	57 (60.6)
Camp (%)	Dagahaley	60 (50.8)	17 (18.7)	77 (36.8)
	Hagadera	58 (49.2)	74 (81.3)	132 (63.2)
NPV^b^ – before Dadaab (%)		19 (16.1)	29 (31.9)	48 (23.0)
IPV^c^ – before Dadaab (%)		15 (12.7)	15 (16.5)	30 (14.4)
Physical IPV^c^ or NPV^b^ – past year (%)		64 (54.2)	63 (69.2)	127 (60.8)
IPV^c^ or NPV^b^ – past year (%)		82 (69.5)	70 (76.9)	152 (72.7)
IPV^c^ – past year (%)		52 (44.1)	46 (50.5)	98 (46.9)
NPV^b^ – past year (%)		41 (34.7)	40 (44.0)	81 (38.8)

a Calculated out of women with a current partner.

b Non-partner violence (NPV): physical and/or sexual violence perpetrated by a non-partner.

c Intimate partner violence (IPV): physical, sexual and/or emotional violence perpetrated by an intimate male partner. IQR, interquartile range; KES, Kenyan Shilling.

Overall, 44% of the women accessing GBV services reported a disability in the baseline cohort survey. Women with a disability were slightly older on average compared to those reporting no disabilities (Table 1). Further, they were more likely to have reported not having a current male partner (60% among women with a disability *v*. 51% without) and to have reported caring for four or more children (45% *v.* 37%). The reported income for women with a disability was higher across all income categories compared to women with no disabilities.

Among all women reporting a disability, the functional disability domains most often reported included difficulties with memory and/or concentration (75%) and difficulties walking (44%). Fewer women reported sensory impairments including vision (9%), hearing (2%), self-care (7%), and communication challenges (1%) (Table 2). The WG-SS demonstrated average internal reliability (Cronbach's *α* = 0.53).

**Table 2:** Prevalence of Washington Group Short Set functional disability domains among refugee women accessing gender-based violence services in a refugee camp

	Response: ‘a lot of difficulty’ or ‘cannot do’	
Prevalence	*N*	% (*N* = 91)^a^
Functional difficulty in the following disability domains:		
Do you have difficulty seeing, even if wearing glasses?	8	9
Do you have difficulty hearing, even if using a hearing aid?	2	2
Do you have difficulty walking or climbing steps?	40	44
Do you have difficulty remembering or concentrating?	68	75
Do you have difficulty (with self-care such as) washing your whole body or getting dressed?	6	7
Using your usual (customary) language, do you have difficulty communicating, for example understanding or being understood?	1	1

a Out of all women identified with a disability.

### Prevalence of violence and disability

Among all participants, 23% reported experience of non-partner physical or sexual violence before arriving in Dadaab and 73% reported experience of physical and/or sexual IPV or NPV in the past year (Table 1). Women in the sample with a disability consistently reported a higher prevalence of experiencing violence before arriving in Dadaab and within the past year than women without a disability. For example, a higher proportion of women with a disability reported experiences of physical or sexual NPV (32%) compared to women without a disability (16%). Further, 69% of women with a disability reported a past-year experience of physical IPV and/or physical or sexual NPV, compared to 54% of women without a disability. Reports of both IPV in the past year (51% *v.* 44%) and NPV in the past year (44% and 35%) were also higher among women with a disability compared to women without a disability.

The internal reliability of these three GBV items varied (past year IPV: Cronbach's *α* = 0.92; past year NPV: Cronbach's *α* = 0.53; before Dadaab NPV: Cronbach's *α* = 0.44).

Logistic regression models exploring the association between reported violence and functional disability suggest that having a disability may be associated with NPV before Dadaab, though the 95% CI for this effect size did not rule out no association (Table 3).

**Table 3:** Modelling the association between reported GBV and functional disability status.

Logistic regression – disability (at baseline)	Crude model			Adjusted model		
	Odds ratio	95% CI	*p* value	Odds ratio	95% CI	*p* value
Past year IPV: yes (ref = no)	1.38	[0.74, 2.57]	0.32	1.91	[0.95, 3.84]	0.07
Past year NPV: yes (ref = no)	1.69	[0.90, 3.17]	0.10	1.73	[0.91, 3.30]	0.10
Any NPV before Dadaab: yes (ref = no)	1.93	[0.95, 3.94]	0.07	2.17	[0.95, 5.01]	0.07
Age (years), mean centred				1.04	[0.99, 1.09]	0.12
Length of encampment (years)				1.06	[1.01, 1.10]	0.01
Number of children				1.03	[0.91, 1.17]	0.59
Partner status: partnered, not living together (ref = no partner)				0.57	[0.25, 1.29]	0.18
Partnered, living together (ref = no partner)				0.63	[0.29, 1.36]	0.24
*N*	204			204		
AIC	263			258		

### Prevalence of mental health outcomes by disability status

Overall, more than a third of women reported symptoms indicating moderate/severe depression (36%), 41% reported symptoms of moderate/severe anxiety and 3% reported symptoms of probable PTSD. Women with a disability reported a substantially higher prevalence of all mental health outcomes – 50% of women with a disability reported symptoms of depression, 52% reported symptoms of anxiety, and 7% reported symptoms of probable PTSD (Table 4).

**Table 4:** Prevalence of anxiety, depression, and PTSD symptoms among refugee women reporting a functional disability

Psychological outcome	No disability *N* = 118, *N* (%)	Disability *N* = 91, *N* (%)	Total *N* = 209, *N* (%)
Moderate/severe anxiety	38 (32.2)	47 (51.6)	85 (40.7)
Moderate/severe depression	30 (25.4)	45 (49.5)	75 (35.9)
Probable PTSD	0 (0.0)	6 (6.6)	6 (2.9)

Within our study population, each of these symptomatic scales demonstrated good internal reliability (anxiety: Cronbach's *α* = 0.77, depression: Cronbach's *α* = 0.77 and PTSD: Cronbach's *α* = 0.83).

### Relationship between mental health, violence, and disability

Among women attending the GBV services, reporting a functional disability was associated with higher symptomatic scores for anxiety (1.54 points higher, 95% CI 0.13–2.95) after adjusting for age, length of the encampment, partner status, number of children, and GBV indicators (Table 5). There was also evidence that reporting a disability is associated with higher scores for depression (1.93 points higher, 95% CI 0.54–3.33) after adjusting for age, length of the encampment, partner status, number of children, and GBV indicators. Disability was also associated with higher scores for PTSD (2.26 points higher, 95% CI 0.03–4.49) after adjusting for other model covariates. After restricting the definition of functional disability to exclude remembering/concentrating (common symptoms of these mental health conditions), these findings remained consistent (see online Supplementary Appendix).

**Table 5:** Modelling the association between anxiety, depression, or PTSD symptomology and reporting a functional disability

Anxiety linear regression model	Unadjusted			Adjusted (not including violence items)			Adjusted (including violence items)		
	Coef	95% CI	*p* value	Coef	95% CI	*p* value	Coef	95% CI	*p* value
Any functional disability: yes (ref = no)	1.77	[0.41, 3.13]	0.01	1.94	[0.56, 3.32]	0.007	1.54	[0.13, 2.95]	0.03
Age (years), mean-centred				−0.08	[−0.17, −0.01]	0.05	−0.11	[−0.20, −0.02]	0.02
Length of encampment (years)				−0.05	[−0.13, 0.03]	0.26	0	[−0.08, 0.09]	0.94
Partner, not living together (ref = no partner)				−0.76	[−2.55, 0.92]	0.41	−0.41	[−2.09, 1.27]	0.64
Partner, living together (ref = no partner)				−1.05	[−2.55, 0.45]	0.17	−0.56	[−2.11, 0.99]	0.48
Number of children				0.23	[−0.02, 0.48]	0.07	0.23	[−0.01, 0.47]	0.08
Past year IPV: yes (ref = no)							0.23	[−1.18, 1.64]	0.75
Past year NPV: yes (ref = no)							0.51	[−0.87, 1.89]	0.47
Any NPV before Dadaab: yes (ref = no)							2.95	[1.27, 4.63]	0.001
*N*	204			204			204		
*R*-squared	0.78			0.78			0.79		
AIC	1205			1207			1198		
RMSE	4.60			4.56			4.44		

Depression linear regression model	Unadjusted			Adjusted (not including violence items)			Adjusted (including violence items)		
	Coef	95% CI	*p* value	Coef	95% CI	*p* value	Coef	95% CI	*p* value
Any functional disability: yes (ref = no)	2.13	[0.81, 3.45]	0.002	2.36	[0.97, 3.75]	0.001	1.93	[0.54, 3.33]	0.01
Age (years), mean centred				−0.03	[−0.12, 0.06]	0.43	−0.03	[−0.11, 0.05]	0.49
Length of encampment (years)				−0.09	[−0.17, ��0.01]	0.03	−0.07	[−0.15, 0.02]	0.14
Partner, not living together (ref = no partner)				−0.80	[−2.43, 0.83]	0.33	−1.05	[−2.85, 0.74]	0.19
Partner, living together (ref = no partner)				−1.17	[−2.70, 0.35]	0.12	−1.28	[−2.80, 0.24]	0.10
Number of children				−0.01	[−0.27, 0.25]	0.94	−0.01	[−0.26, 0.24]	0.94
Past year IPV: yes (ref = no)							1.60	[0.24, 2.95]	0.02
Past year NPV: yes (ref = no)							1.07	[−0.26, 2.41]	0.11
Any NPV before Dadaab: yes (ref = no)							1.54	[−0.20, 3.28]	0.07
*N*	204			204			204		
*R*-squared	0.77			0.77			0.78		
AIC	1198			1199			1195		
RMSE	4.52			4.48			4.40		

PTSD linear regression model	Unadjusted			Adjusted (not including violence items)			Adjusted (including violence items)		
	Coef	95% CI	*p* value	Coef	95% CI	*p* value	Coef	95% CI	*p* value
Any functional disability: yes (ref = no)	3.04	[0.88,5.19]	0.006	3.07	[0.86, 5.29]	0.007	2.26	[0.03, 4.49]	0.05
Age (years), mean centred				−0.02	[−0.14, 0.10]	0.75	−0.07	[−0.20, 0.05]	0.27
Length of encampment (years)				−0.15	[−0.27, −0.02]	0.04	−0.05	[−0.18, 0.08]	0.53
Partner, not living together (ref = no partner)				−2.32	[−4.74, 0.11]	0.12	−1.60	[−4.06, 0.86]	0.20
Partner, living together (ref = no partner)				−3.56	[−6.13, −0.98]	0.007	−2.59	[−5.14, −0.04]	0.04
Number of children				0.16	[−0.23, 0.54]	0.42	0.16	[−0.19, 0.51]	0.37
Past year IPV: yes (ref = no)							0.41	[−1.81, 2.62]	0.72
Past year NPV: yes (ref = no)							1.26	[−0.93, 3.45]	0.24
Any NPV before Dadaab: yes (ref = no)							5.71	[3.15, 8.26]	<0.001
*N*	204			204			204		
*R*-squared	0.80			0.81			0.82		
AIC	1405			1401			1385		
RMSE	7.51			7.35			7.03		

AIC, Akaike information criterion; IPV, intimate partner violence; NPV, non-partner violence; RMSE, root-mean-square error.

We found strong evidence that an experience of NPV before arriving in the Dadaab refugee camps was associated with higher scores for anxiety (2.95, 95% CI 1.27–4.63) and probable PTSD (5.71, 95% CI 3.15–8.26) (Table 5).

## Discussion

The prevalence of disability among refugee women accessing GBV response services in the Dadaab refugee camps was high – with nearly half of all women surveyed (44%) being classified as disabled. This figure for disability prevalence is higher than would be expected for a population of this age group. Our findings are consistent with other research from non-camp settings showing that people with disabilities are at increased risk of violence and exploitation (Mirza, 2015; Dunkle *et al*., 2018; Scolese *et al*., 2020a). Our research also suggests that refugee women with a disability are more likely to report poor mental health conditions (depression, anxiety, and PTSD), which is consistent with the existing literature (Steel *et al*., 2009; Bogic *et al*., 2015; Abu Suhaiban *et al*., 2019). In addition, conflict-related violence and other NPV which occurred before arriving in the Dadaab refugee camps continued to have a long-term impact on women's mental health – women who reported NPV before arriving in Dadaab had higher levels of depression and PTSD. A sensitivity analysis demonstrated that our findings remained consistent when the definition of functional disability was restricted to exclude women with depression symptomology (i.e. difficulty remembering/concentrating).

Our findings are consistent with other research conducted in non-humanitarian settings. Our study extends these findings and importantly adds to the evidence base as one of the first to examine the levels of disability and mental health conditions among a cohort of refugee women accessing GBV response services in a camp setting. There is limited evidence examining the intersection of disability, violence, and mental health within GBV services in a refugee camp setting. Our research findings highlight the importance of having refugee camp services that are accessible to and address the mental health needs of, refugees with disabilities who have experienced violence. Other research has found that people with disabilities may be at increased risk of poor mental health, due to lack of social support, extreme marginalisation, stigma, discrimination, and additional barriers to accessing health and social services (Ganle *et al*., 2020; Stern *et al*., 2020). Therefore, the implications for our findings are primarily programmatic – women with disabilities who have experienced violence must be able to access GBV response services and these services must address their mental health needs. This can be accomplished through screening for mental health and disability combined with disability-inclusive outreach within GBV response programming to ensure that case workers can effectively address the needs of survivors with disabilities and incorporate necessary rehabilitation or specialised services.

Our findings also suggest that women with more severe disabilities are not reaching GBV response services. Women with more severe physical or cognitive disabilities did not access the GBV services, suggesting that they may have faced challenges getting to theservices. All women enrolled in the cohort study reported challenges accessing services, especially due to the on-going camp closure and repatriation processes that were underway during the cohort study (Hossain *et al*., 2018; Muuo *et al*., 2020). Therefore, it is feasible that women with more severe disabilities faced significant difficulties accessing GBV response services within the camp. There is a need to expand outreach activities to ensure that women and girls at higher risk of violence and discrimination have access to services on an equal basis and without barriers. One promising way to achieve this is through task-sharing with refugee community workers who have been trained to help improve access to refugees with disabilities (Hossain *et al*., 2018). GBV response programmes can also improve access by engaging with local disability actors to increase promotion and access to services. Other research has highlighted the challenges of delivering IPV programming for people with disabilities in developing countries (Dunkle *et al*., 2018; Stern *et al*., 2020).

Several limitations need to be considered when evaluating the findings. Due to logistical, budgetary, and ethical constraints, we were unable to survey a control group, therefore, we are unable to assess differences with women who did not access GBV response services. In addition, this study was designed to recruit at least 400 women; however, only 209 women were enrolled in the study due to threats of a camp closure and repatriation that coincided with the start of the cohort study. Our analyses, therefore, had limited power to detect associations within sub-sets of the data including whether disability moderated the relationship between our GBV measures and mental health symptomology (and vice versa). In particular, we are unable to examine the association of violence and mental health among older women and adolescent girls with disabilities as our cohort did not include anyone under 18, and few women were older than 45 years old (*n* = 8). For ethical and safety reasons, women were screened by professional caseworkers before being invited to participate in the study. Women with intellectual disabilities, psychosocial disabilities, and who were experiencing severe GBV may have been assessed by the caseworker as unable to participate without causing further trauma and were not enrolledin the study. This would have led to underestimations in the associations between disability and mental health symptomology.

The WG-SS of questions used to measure disability does not include mental health conditions, therefore, women with psychosocial disabilities are also likely to be under-detected in our cohort. There were also few women who reported sensory impairments related vision or hearing which could indicate that GBV services were not accessible to women with some types of disabilities. The study was focused on women accessing services; therefore, we do not know the prevalence of disability among women who are not attending GBV services.

Violence can worsen an existing disability or lead to a new impairment, including mental health conditions such as anxiety, depression, and PTSD. It is, therefore, possible that GBV acted as a mediator or effect modifier in our models; however, we were unable to determine the temporal nature of the associations between disability, violence, and mental health. Further, due to the limited sample size and cross-sectional data, we are unable to conclude if depression, PTSD, or anxiety contributed to the types of disabilities reported or increased the risk of experiencing violence.

Finally, our survey did not capture all experiences of GBV as 27% of all women accessing services reported no past-year GBV experience and 12% reported no lifetime GBV experience. Free-text fields to record the reason of the service visit revealed that many of these women accessed the GBV services in response to threats and intimidation from within the community or their own families. However, the survey did not capture emotional distress caused by non-partners, which may explain why some women appear to have no lifetime experiences of GBV in the study data, although they were eligible to receive services. In addition, violence against people with disabilities can include restriction of movement, denial of access to assistive devices or services, forced medical treatment (including forced sterilisation or use of drugs), and other forms of deprivations of rights (Dunkle *et al*., 2018; Stern *et al*., 2020): questions about which were not included in our survey. There were also low reports of sexual violence among women enrolled in the cohort. Although they attended the GBV services voluntarily and interviews were conducted in a safe environment, it is likely that the stigma attached to sexual violence, in particular, its implications on a woman's perceived ‘honour’, led to under-reporting of this particular subtype of violence. In addition, it is probable that recall bias affected reporting for some acts of historical violence.

## Conclusion

This study fills an important evidence gap in understanding the service delivery needs of women accessing GBV response services within refugee camp settings. Disability is a key factor in any intersectional analysis completed by GBV actors to inform inclusive GBV programming. GBV actors should consider barriers and risks facing diverse women and girls on the basis of their gender, age, socio-economic status, race, ethnicity, religion, language, sexual orientation, gender identity, and other locally relevant factors. Actions to include women and girls with disabilities within GBV programming should not be a standalone or separate activity but an inherent part of quality GBV programming. A survivor-centred, feminist approach to implement GBV programming is well-aligned with disability activism and minimum standards for GBV programming in humanitarian settings (Inter-Agency Standing Committee (IASC), 2015; IASC Task Team on Inclusion of Persons with Disabilities in Humanitarian Action, 2019). Both promote programming which is led by, and accountable to, women and girls with disabilities; focuses on the inherent strengths of diverse women and girls; and calls out the systemic inequality-based gender and disability which undermines the rights of women and girls with disabilities to safe and equitable access to humanitarian aid and to pursue their potential, free from violence and inequality.

Further research is urgently needed within humanitarian settings to understand the extent of violence against women, men, and children with disabilities and how to effectively provide programming that prevents and responds to various forms of gendered and discriminatory violence. GBV programming must seek ways to become more inclusive so that persons with disabilities are able to access and utilise psychosocial, medical, justice, and other needed services.

## Data

The datasets analysed during the current study are available in the LSHTM COMPASS data repository, https://datacompass.lshtm.ac.uk/1511 on reasonable request.
